# Compliance to contact lens wear and care among Jordanian adults

**DOI:** 10.1371/journal.pone.0280409

**Published:** 2023-01-11

**Authors:** Yazan Gammoh, Wafa Asfour

**Affiliations:** Faculty of Allied Medical Sciences, Department of Optometry Science, Al-Ahliyya Amman University, Amman, Jordan; The Ohio State University, UNITED STATES

## Abstract

**Objective:**

To evaluate compliance to contact lens (CL) wear and care routines among adults in Jordan.

**Design:**

A cross-sectional study using a questionnaire administered by an interviewer was conducted among adults in Jordan between the months of May and July of the year 2022.

**Participants:**

Adult (≥ 18 years) contact lens wearers attending work establishments, universities and shopping centers in the cities of Amman, Aqaba and Irbid in Jordan.

**Main outcomes and measures:**

Compliance to CL wear and care in addition to CL hygiene habits were evaluated.

**Results:**

A total of 834 (600 women) participants were included in the study with an age range of 18 to 55 years (average; 25.49 ± 7.75). Soft spherical CLs for myopia correction were worn by 45.6% of the sample, followed by cosmetic CLs (CCL) (43%). Highest compliance rate (99%) was calculated for not sharing CL with others and the lowest rate (24%) was for cleaning the CL case, with an average compliance rate of 72.25% for the sample with all habits surveyed. Medium compliance rates were related to following instructions of CL solution use including avoiding using expired solution or avoiding topping off. Risk factors for non-compliant behaviors included CCL wear, purchasing CL from beauty centers and being 25 years of age or younger.

**Conclusions and relevance:**

This study which was performed in a non-clinical setting showed that women and young adults comprise the majority of CL wearers in Jordan. Participants were compliant with most CL wear and care behaviors except for cleaning the CL case and attending aftercare visits. Many CL wearers also reported purchasing lenses from beauty centers and online without consulting CL practitioners. There is a need for patient education regarding the hygiene of the CL case, and the necessity for consulting the CL practitioner for the appropriate type of CL including proper wear and care regimen.

## Introduction

Myopia is the most common refractive error of the eye, with almost half of the world population expected to be myopic by the year 2050 [1]. The financial impact of myopia on public health is considered high due to the cost associated with the optical correction of myopia which includes spectacles and contact lenses (CLs) [2]. Besides spectacles, CLs are considered a popular choice for correcting refractive errors, including myopia [3]. Other uses of CLs include cosmesis in the form of cosmetic contact lenses (CCLs) and keratoconus correction, mainly using gas-permeable contact lenses (GP) [4–6]. In the Middle East, CCLs and soft CLs for myopia correction are the most common CLs used or prescribed [7–11]. However, since available literature on CLs in the Middle East has focused on university students [8,9], and women, or collecting information from settings such as beauty centers [10], it may not truly represent the CL wear pattern in the general adult population [11].

The sale of CLs without a prescription or purchasing CLs from online resources, beauty centers and pharmacies is widely practiced in Middle Eastern and other developing countries [8–14]. It is not feasible to understand or assess the level of compliance demonstrated by CL wearers when users purchase their CLs from a non-clinical setting. Practitioners exacerbate this by not promoting CL wear as an option for refractive error correction or providing advice on the use of CLs and their care products [8,13,14]. Reported non-compliance to CL wear and care in the Middle East and other developing countries encompasses several behaviors such as inadequate cleaning and replacement of CL case, not adhering to CL cleaning instructions, and not attending aftercare visits to the CL practitioner [8–11,13,14]. These non-compliant behaviors are related to CL user characteristics such as age, gender and smoking habit [15–20]. With limited availability of information on contact lens wear and care compliance from the general adult population, the study aimed to investigate compliance levels and factors that contribute to non-compliant behaviors among adult CL wearers in Jordan.

## Materials and methods

A cross-sectional study using a questionnaire administered by an interviewer was conducted among adults (≥18 years) in Jordan between the months of May and July of the year 2022. The questionnaire (S1 Table) has been used previously in literature to investigate adult CL wearers’ compliance to wear and care of CL, in addition to hygiene behaviors [8,9,14]. The questionnaire included questions about the type of CL used and the CL care product. It has been suggested that photographs of products be shown to participants who were not able to recall the products they use, thus photos of CLs brands and types and CL care products were used in this study [14,21]. Participants were recruited from work establishments, universities and shopping centers in the cities of Amman, Aqaba and Irbid in Jordan. This sampling technique has been used to assess the prevalence of refractive errors among adults in Jordan [22]. To avoid sampling bias, a systematic sampling method was used where every third person entering the establishment or passing the main hall of a building was approached. The study was conducted during all working days of the establishments at the time between 9:00 and 11:00 am, and between 1:00 and 3:00 pm before the end of the working day. When approaching persons in shopping centers, participants were approached at the same times mentioned above in addition to an extra session between 5:00 pm and 7:00 pm as well as on weekends to ensure that a variety of the population would be encountered. Only adult Jordanians who had been wearing CLs for over a month, and willing to participate and understood the nature of the questions were included in the study [14,21]. To avoid inter-user sampling error, the participants were interviewed by the same researcher (YG) to complete the contact lens compliance questionnaire.

Participants who reported active ocular pathology were excluded from the study [8,14]. Written informed consent was obtained from the participants and tenets of the Declaration of Helsinki were followed. Ethical approval (AAU-FHS-21/22) was obtained from the Faculty of Allied Medical Sciences, Al-Ahliyya Amman University. As the study aimed to include adults who wore all CL types, including cosmetic lenses, rather than just adults with refractive errors, the Jordanian adult population size was used to collect the sample size [14,23]. Using GRANMO version 7.12 and based on a population size of approximately 6,000,000 Jordanian adults who are at least 18 years old; a sample size of 405 was considered representative [14,24]. Confidence interval level was set at 95%, with a ± 5% margin of error and a replacement rate of 5%. The methods followed in this study and the results obtained followed the recommendations set by the Strengthening of Reporting in Observational studies in Epidemiology (STROBE) statement (S2 Table) [25].

As previously reported in the literature, responses to questions were recorded as scores from 1 to 4, with scores 1 and 2 indicating compliance, while scores 3 and 4 indicated non-compliance [8,9,14]. Scores ranging from 1 for (rarely) to 4 for (always) were recorded for the following behaviors: Sleeping or napping with CLs, sharing CLs, showering and swimming while wearing CLs, sharing the CL solution bottle, sharing the CL case, topping off the solution and rinsing CLs with tap water. Scores from 1 to 4 were recorded for responses ranging from (Always) to (Rarely) for the following behaviors: checking expiry date of solution, use of enough solution in CL case, washing hands prior to insertion and removal of CLs, adherence to corrects steps while cleaning and storing CLs, and cleaning the lens case. Replacement of CL case after 1 month was scored as (1), replacement every 3 months as (2), replacement after 6 months as (3), and score 4 was assigned for over 6 months. Microsoft Excel Spreadsheets were used to enter data and scores, while SPSS software version 25 (IBM Corporation, Armonk, NY, USA) was utilized for data analysis. Nominal and categorical data were presented as numbers and percentages. Mann-Whitney U-test and Kruskal-Wallis test were utilized to investigate non-compliance related factors. Statistical significance was set at a P value lower than 0.05.

## Results and discussion

The number of participants included in the analysis was 834, of whom 600 (71.9%) were women. Participants’ age ranged between 18 and 55 years with an average of 25.49 ± 7.75. Table 1 details participants’ demographics including refractive error distribution.

**Table 1:** Demographics of participants (n = 834).

Demographic factor	Number (%)
**Age (years)** 18–25 26–35 36–45 46–55	631 (75.5%) 113 (13.5%) 45 (5.5%) 45 (5.5%)
**Gender** Women Men	600 (71.9%) 234 (28.1%)
**Region of residence** North Middle South	131 (15.7%) 630 (75.5%) 73 (8.8%)
**Education** High school Undergraduate Postgraduate	51 (6.1%) 724 (86.8%) 59 (7.1%)
**Occupation** Clerical Professional Skilled Students Unemployed Retired	90 (10.8%) 276 (33.1%) 78 (9.4%) 368 (44.1%) 17 (2%) 5 (0.6%)
**Smoking** No Yes	550 (66%) 284 (34%)
**Refractive error** None Hyperopia Myopia Astigmatism Keratoconus	361 (43.3%) 13 (1.6%) 372 (44.6%) 62 (7.4%) 26 (3.1%)

### Profile of CL wear

Table 2 depicts the profile of CL wear among the sample population. Soft multifocal CLs were the least utilized type of CLs used among the sample population. CLs replaced quarterly (every 3 months) were the least popular. Most of the participants reported wearing CLs for 12 months or less. Multipurpose CL solutions use was reported by most of the sample population. Participants reported wearing CLs for an average of 6.78 (± 1.60) hours per day, with a CL wear experience of an average of 19.81 (± 16.24) months.

**Table 2:** Profile of contact lens (CL) wear (n = 834).

Information	Category	Number (%)
**CL type**	Soft spherical Soft toric Soft cosmetic Soft multifocal Rigid-gas-permeable	380 (45.6%) 54 (6.5%) 361 (43.3%) 5 (0.6%) 34 (4%)
**CL power**	Plano ≤5 Diopter >5 Diopter	361 (43.3%) 428 (51.3%) 45 (5.4%)
**CL experience**	≤ 12 months 13–24 month 25–36 month 37–48 month >48 month	439 (52.7%) 218 (26.1%) 82 (9.8%) 39 (4.7%) 56 (6.7%)
**CL wearing time per day**	1–5 hours 6–11 hours ≥ 12 hours	137 (16.5%) 680 (81.5%) 17 (2%)
**CL wearing modality**	Daily disposable Monthly replacement Quarterly replacement Yearly replacement	221 (26.5%) 495 (59.4%) 8 (0.9%) 110 (13.2%)
**Place of CL purchase**	Contact lens practitioner Pharmacy Internet Beauty center	498 (59.7%) 78 (9.4%) 174 (20.9%) 84 (10%)
**CL care solution**	Hydrogen peroxide system Multipurpose solution Do not use solution	14 (1.7%) 599 (71.8%) 221 (26.5%)

### Compliance to CL wear and care behaviors

Table 3 details scores from 1 to 4 for positive CL wear and care behaviors. A score of 1 indicates that the participant is strongly compliant, score 2 means the participant is compliant. Score 3 indicates that the participant is non-compliant while score 4 means that the participant is strongly non-compliant.

**Table 3:** Level of compliance to positive behaviors associated with contact lens (CL) wear and care (n = 834).

Behavior	Score 1* n (%)	Score 2* n (%)	Score 3* n (%)	Score 4* n (%)
Hands washing before inserting CL	538 (64.5)	141 (16.9)	64 (7.7)	91 (10.9)
Cleaning lens case	153 (18.3)	43 (5.2)	314 (37.6)	324 (38.9)
Rubbing, rinsing & soaking with prescribed solution	411 (49.3)	48 (5.7)	189 (22.7)	186 (22.3)
Lens case replacement	408 (48.9)	161 (19.3)	174 (20.9)	91 (10.9)
Attending after-care visits	187 (22.4)	124 (14.9)	360 (43.2)	163 (19.5)

*score 1: Strongly compliant, score 2: Compliant, score 3: Non-compliant, and score 4: Strongly non-compliant.

Table 4 details scores to from 1 to 4 for negative CL wear and care behaviors. A score of 1 indicates that the participant is strongly compliant, score 2 means the participant is compliant. Score 3 indicates that the participant is non-compliant while score 4 means that the participant is strongly non-compliant.

**Table 4:** Level of compliance to negative behaviors associated with contact lens (CL) wear and care (n = 834).

Behavior	Score 1* n (%)	Score 2* n (%)	Score 3* n (%)	Score 4* n (%)
Showering/swimming with CL	558 (66.9)	151 (18.1)	45 (5.4)	80 (9.6)
Sharing CL with others	722 (86.5)	105 (12.6)	4 (0.5)	3 (0.4)
Sleeping/napping with CL	561 (67.3)	234 (28)	35 (4.2)	4 (0.5)
Usage beyond recommended replacement schedule	221 (26.5)	504 (60.5)	107 (12.8)	2 (0.2)
Use solution after expiry date	478 (57.3)	97 (11.6)	103 (12.4)	156 (18.7)
Topping off solution	471 (56.5)	143 (17.1)	152 (18.2)	68 (8.2)
Rinsing lens with tap water	607 (72.8)	168 (20.1)	59 (7.1)	0 (0)

*score 1: Strongly compliant, score 2: Compliant, score 3: Non-compliant, and score 4: Strongly non-compliant.

### Risk factors for non-compliant CL wear and care behavior

Table 5 shows the factors that were significantly associated with non-compliant behaviors among the sample population. CCLs wear, plano-lens prescriptions, smoking and being between 18 and 25 years of age were common factors in multiple non-compliant behaviors.

**Table 5:** Risk factors significantly associated with non-compliant behaviors among Jordanian adult contact lens (CL) wearers.

Non-compliant Behavior	Factor leading to poor compliance	P value
Showering/swimming with CL	• Multifocal CL wear • Women • Cosmetic CL wear	0.01 <0.001 <0.001
Sharing CL with others	• Plano prescription • Purchase CL from beauty center	<0.001 <0.001
Sleeping/napping with CL	• Purchase CL from beauty center • Age group (46–55) • Hyperopia	0.02 <0.001 <0.001
Usage beyond recommended replacement schedule	• Age group (18–25) • Keratoconus • Prescription >5 • Rigid Gas permeable CL wear • Yearly CL replacement • CL wear experience >48 months	0.01 <0.001 <0.001 <0.001 <0.001 <0.001
Topping off the solution	• Undergraduate degree • CL wear 1–5 hours per day • Men • Age group (18–25) • Purchase CL from internet	0.02 0.005 <0.001 <0.001 <0.001
Not Checking the expiry date of the solution	• Men • Soft CL wear	<0.001 <0.001
No Hand washing before inserting CL	• Purchase CL from beauty center	0.004
Rinsing lens with tap water	• Women • Cosmetic CL wear • Keratoconus • Prescription >5	0.023 <0.001 <0.001 <0.001
Not rubbing, rinsing & soaking with the prescribed solution	• Men • Smokers	0.015 0.002
Not Cleaning the CL case	• Smokers • Cosmetic CL wear	<0.001 <0.001
Infrequent Lens case replacement	• Men • Cosmetic CL wear	0.024 <0.001
Not attending after care visits	• CL wear ≥ 12 hours per day • Age group (18–25) • Smokers • Men • Cosmetic CL wear • Plano prescription • Quarterly CL replacement	0.03 0.036 0.004 <0.001 <0.001 <0.001 <0.001

This study established the profile of CL wear and care, in addition to compliance levels and factors related to non-compliant behavior among a sample of adult CL wearers in Jordan from a non-clinical setting. Only one study reported compliance with CL wear in Jordan where data was derived from university students who attend the campus CL clinic which does not allow for understanding the CL compliance behaviors among the general Jordanian adult population [8]. Despite the attempt of the current study to collect data from the general Jordanian adult population using systematic sampling from non-clinical settings such as shopping centers and work establishments, women constituted the majority of the sample population which agrees with reports from Jordan and other Middle Eastern and developing countries [7–14]. In addition, the majority of the sample population were young adults of an average age of approximately 25 years which is in alignment with the average age of adults fitted with contact lenses in clinical practices in Jordan [7].

Soft spherical CLs worn monthly for myopia correction are the most common type and modality of CLs worn by the participants. This is in agreement with the trends of CL prescribing in Jordan and the prevalence of myopia (53.7%) among adult Jordanians [7,22]. The least used type of CLs by the sample population was RGPs and they were worn exclusively to correct keratoconus and high astigmatism. This is in agreement with previous studies where the use of RGP CLs remains low among CL wearers [6,7,14,26]. Multifocal CLs were the least utilized type (0.6%) of CLs by the sample population. A previous study about trends of CL prescribing in Jordan reported that multifocal CLs are minimally used due to the young age of CL wearers or perhaps the lack of awareness of CL users about the availability of multifocal CLs [6]. In addition, the scope of practice of optometrists and the limited availability of resources may not allow for comprehensive CL provision to the community [12,27]. In terms of CL care products, multipurpose solutions remain the most popular CL solution as reported previously among CL wearers in Jordan potentially due to the availability and affordable cost of this class of solutions [7,8]. Apart from CL practitioners, pharmacies, beauty centers and online sites were sources of CL purchasing by the sample population. This is in agreement with trends of CL purchasing in the Middle East [8–11].

Strong compliance was observed among the sample population for not wearing CLs when showering or swimming, and for handwashing before wearing CLs which is in contrast to data available from university students in Jordan [8]. CCLs and multifocals wear were factors related to non-compliance related to showering and swimming with CLs. As CCLs in the Middle East, including Jordan, are not considered medical devices and are not prescribed by CL practitioners or are usually sold in beauty centers [10,14,28], users may not be aware of the risk of CL contamination from water which may lead to microbial keratitis [29]. A strong compliance rate was also observed for the habits of avoiding sleeping in CLs and avoiding usage beyond recommended schedule. The highest compliance rate was observed for avoiding sharing CLs with others which was also reported previously among Jordanian university students [8]. In the current sample population, 44.1% were university students, which is in agreement with the population composition in Jordan [23], and would explain the similarities between the current study and the literature available.

Participants were mainly compliant with behaviors related to the use of CL care products such as avoiding topping off the CL solution (adding fresh solution to existing solution in lens case), and avoiding using CL solution beyond expiry date. Men, young age (less than 26 years) and wear time less than six hours per day were risk factors for non-compliance with the above-mentioned behaviors. Gender was not observed to be a factor in non-compliance among university students in Jordan [8]. Though men have been reported to be non-compliant in other countries [8,30], the role of gender in CL compliance is not clearly understood [8,30]. The role of age in CL compliance is also not well demonstrated since most data available are from university students with a limited age range [8,14].

Attendance to aftercare visits and cleaning the CL case showed the lowest compliance rates in the sample population. Such non-compliance was observed in several other studies among university students and the general adult population [8,9,14]. Improper cleaning of the CL case has been demonstrated to cause microbial contamination of CLs despite the proper cleaning of CLs [31,32]. Smoking was observed to be a factor in non-compliance with CL case cleaning and attending after-care visits, which is in agreement with previous studies [8,14]. Smoking is not advised with CLs as it may increase the risk of microbial keratitis which could be detrimental to the health of the eye [33].

### Conclusions, limitations and recommendations

Adult CL wearers recruited in the study were mostly women, in the second decade of their age, and wore monthly soft CLs for myopia correction. CCLs are also a popular choice which are mostly worn as daily disposables and usually purchased from beauty centers. Compliance was observed with most CL wear and care habits except for cleaning the CL case and attendance to aftercare visits. Smoking, wearing CCLs, and being under the age of 26 years are the main factors that were significantly associated with non-compliant behaviors.

This study was not conducted in a clinical setting to avoid false high compliance perceived by CL wearers when attending the clinic [12,19,21]. Collecting data from adults attending various work settings, universities and shopping centers has been previously attempted in the Middle East [11,22]. Nevertheless, non-randomness and selection bias could have been introduced due to the socio-demographic profile of CL wearers attending such settings, though shopping centers, mainly the supermarkets within, are frequented by Jordanians with various socio-economic backgrounds which would indicate minimum selection bias. Despite employing a systematic sampling method while collecting data from persons attending various working establishments to avoid gender or age selection bias, the sample was mostly comprised of women. One of the limitations of the study is inferring the demographics of contact lens wearers from the individuals who participated in the study. It is recommended that a log be kept of every potential who was approached during the data collection phase in order to make assumptions about contact lens wear among the overall population of a country. In addition, the sample population were mainly young adults with an average age of around 25 years. The current study shows that adult CL wearers in Jordan are mainly women and young adults. A potential error in reporting the CL wear profile among the sample population due is the inherent inability of the participants to recollect accurately some information related to their CLs. An attempt was made to minimize this by showing photos of CL products to allow participants to better recall the type of CL or CL care product used.

This study represents the first report on CL compliance from adults in Jordan with different age, and socio-economic levels taken from work place settings in all three regions of the country. The data reported in the current study would be valuable to practitioners in terms of understanding the habits of CL wear and care among Jordanian adults and how practitioners should approach non-compliant behavior to ensure safe wear of CLs. Specifically, there is a need for CL prescribing professionals to educate the patients about proper lens wear and care routines. In addition, as this study surveyed established CL wearers, there is a need for CL prescribing professionals to re-educate current CL wearers on compliant habits.

## Supporting information

S1 Table Compliance to contact lens wear and care questionnaire. (DOCX)

S2 Table STROBE statement checklist. (DOC)

S1 Data (XLSX)

## References

[1] Holden BA, Fricke TR, Wilson DA, Jong M, Naidoo KS, Sankaridurg P, et al. Global prevalence of myopia and high myopia and temporal trends from 2000 through 2050. Ophthalmology 2016;123:1036–1042. doi: 10.1016/j.ophtha.2016.01.006 26875007

[2] Rein DB, Zhang P, Wirth KE, Lee PP, Hoerger TJ, McCall N, et al. The economic burden of major adult visual disorders in the United States. Arch Ophthalmol 2006;124:1754–1760. doi: 10.1001/archopht.124.12.1754 17159036

[3] Swanson MW. A cross-sectional analysis of U.S. contact lens user demographics. Optom Vis Sci 2012;89:839–848. doi: 10.1097/OPX.0b013e318255da45 22544000

[4] Rah MJ, Schafer J, Zhang L, Chan O, Roy L, Barr JT. A meta-analysis of studies on cosmetically tinted soft contact lenses. Clin Ophthalmol 2013;7:2037–2042. doi: 10.2147/OPTH.S51600 24143071 PMC3798236

[5] Berenson AB, Chang M, Hirth JM, Merkley KH. Use and misuse of cosmetic contact lenses among US adolescents in Southeast Texas. Adolesc Health Med Ther 2019;10:1–6. doi: 10.2147/AHMT.S196573 30799964 PMC6369860

[6] Morgan P, Woods CA, Tranoudis IG, Efron N, Jones L, Merchan BNL, et al. International contact lens prescribing in 2021. Contact Lens Spectr 2022;37: 32–38.

[7] Haddad MF, Bakkar M, Gammoh Y, Morgan P. Trends of contact lens prescribing in Jordan. Cont Lens Anterior Eye 2016;39:385–388. doi: 10.1016/j.clae.2016.06.004 27364560

[8] Bakkar MM, Alzghoul EA. Assessment of compliance with contact lens wear and care among university-based population in Jordan. Cont Lens Anterior Eye 2020;43:395–401. doi: 10.1016/j.clae.2020.02.020 32127286

[9] Naaman NK, Alharbi SY, Khan MA, Alghamdi SA. Compliance with contact lens care and factors driving noncompliance in health-care students in Jeddah, Saudi Arabia. Saudi J Ophthalmol. 2022;36:75–82. doi: 10.4103/SJOPT.SJOPT_202_21 35971483 PMC9375456

[10] Abahussin M, AlAnazi M, Ogbuehi KC, Osuagwu UL. Prevalence, use and sale of contact lenses in Saudi Arabia: survey on university women and non-ophthalmic stores. Cont Lens Anterior Eye 2014;37:185–190. doi: 10.1016/j.clae.2013.10.001 24211011

[11] Alobaidan OS, Alkhalifah MK, AlSayegh AA, Alhumaid FA, Ashammery AS, Alghamdi K, et al. Knowledge and practice regarding contact lens among Saudi urban contact lens users. Saudi J Ophthalmol 2018;32:93–96. doi: 10.1016/j.sjopt.2017.09.008 29942175 PMC6010592

[12] Boadi-Kusi SB, Ntodie M, Mashige KP, Owusu-Ansah A, Antwi Osei K. A cross-sectional survey of optometrists and optometric practices in Ghana. Clin Exp Optom 2015;98:473–477. doi: 10.1111/cxo.12291 25944332

[13] Abokyi S, Manuh G, Otchere H, Ilechie A. Knowledge, usage and barriers associated with contact lens wear in Ghana. Cont Lens Anterior Eye 2017;40:329–334. doi: 10.1016/j.clae.2017.05.006 28533022

[14] Gammoh Y, Abdu M. (2021). Contact lens procurement and usage habits among adults in Sudan PloS One 2021;16: e0251987. doi: 10.1371/journal.pone.0251987 34010356 PMC8133405

[15] Lievens CW, Cilimberg KC, Moore A. Contact lens care tips for patients: an optometrist’s perspective. Clin Optom (Auckl) 2017;9:113–121. doi: 10.2147/OPTO.S139651 30214367 PMC6118862

[16] Hind J, Williams O, Oladiwura D, Macdonald E. The differences between patient and optometrist experiences of contact lens hygiene education from the perspective of a Scottish university teaching hospital. Cont Lens Anterior Eye 2020;43:185–188. doi: 10.1016/j.clae.2019.07.006 31327578

[17] Robertson DM, Cavanagh HD. Non-compliance with contact lens wear and care practices: a comparative analysis. Optom Vis Sci 2011;88:1402–1408. doi: 10.1097/OPX.0b013e3182333cf9 21946785 PMC3223553

[18] Dumbleton KA, Spafford MM, Sivak A, Jones LW. Exploring compliance: a mixed-methods study of contact lens wearer perspectives. Optom Vis Sci 2013;90: 898–908. doi: 10.1097/OPX.0b013e3182956c46 23770653

[19] Morgan PB, Efron N, Toshida H, Nichols JJ. An international analysis of contact lens compliance. Cont Lens Anterior Eye 2011;34:223–228. doi: 10.1016/j.clae.2011.08.001 21868279

[20] Wu YT, Willcox M, Zhu H, Stapleton F. Contact lens hygiene compliance and lens case contamination: A review. Cont Lens Anterior Eye 2015;38:307–316. doi: 10.1016/j.clae.2015.04.007 25980811

[21] Rueff EM, Wolfe J, Bailey MD. A study of contact lens compliance in a non-clinical setting. Cont Lens Anterior Eye 2019;42:557–561. doi: 10.1016/j.clae.2019.03.001 30890305 PMC6746612

[22] Mallen EA, Gammoh Y, Al-Bdour M, Sayegh FN. Refractive error and ocular biometry in Jordanian adults. Ophthalmic & physiological optics: the journal of the British College of Ophthalmic Opticians (Optometrists) 2005;25:302–309. doi: 10.1111/j.1475-1313.2005.00306.x 15953114

[23] Department of Statistics. Population and Housing 2015. http://dosweb.dos.gov.jo/censuses/population_housing/census2015/; 2015 [accessed 20 May 2022].

[24] GRANMO. Sample size and power calculator. https://www.imim.cat/ofertadeserveis/software-public/granmo/; 2012 [accessed 10 August 2022].

[25] STROBE Statement. Strengthening the reporting of observational studies in epidemiology. https://www.strobe-statement.org/index.php?id=strobe-home; 2014 [accessed 12 August 2022].

[26] Bakkar MM, Haddad MF, Qadire MA. Patient-related barriers to Rigid Gas Permeable (RGP) lens wear among keratoconus patients in Jordan. Cont Lens Anterior Eye 2018;41:267–272. doi: 10.1016/j.clae.2017.12.007 29217454

[27] Okasheh-Otoom A, Gammoh Y, Otoum M, Naqaweh A. The Scope of Optometry Practice in Jordan. Optom Vis Sci. 2022;99:35–44. doi: 10.1097/OPX.0000000000001823 34882601

[28] Medical Devices Registration. Jordan Food and Drug Administration. http://www.jfda.jo/Pages/viewpage.aspx?pageID=364; 2022 [accessed 18 December 2022].

[29] Efron N, Morgan PB. Rethinking contact lens aftercare. Clin Exp Optom. 2017;100:411–431. doi: 10.1111/cxo.12588 28871604

[30] Supiyaphun C, Jongkhajornpong P. Contact Lens Use Patterns, Behavior and Knowledge Among University Students in Thailand. Clin Ophthalmol 2021;15:1249–1258. doi: 10.2147/OPTH.S304735 33790532 PMC8005268

[31] Wu YT, Zhu H, Harmis NY, Iskandar SY, Willcox M, Stapleton F. Profile and frequency of microbial contamination of contact lens cases. Optom Vis Sci 2010;87:E152–158. doi: 10.1097/OPX.0b013e3181cf86ee 20101194

[32] Tilia D, Lazon de la Jara P, Zhu H, Naduvilath TJ, Holden BA. The effect of compliance on contact lens case contamination. Optom Vis Sci 2014;91:262–271. doi: 10.1097/OPX.0000000000000163 24413272

[33] Stapleton F, Edwards K, Keay L, et al. Risk factors for moderate and severe microbial keratitis in daily wear contact lens users. Ophthalmology. 2012;119:1516–1521. doi: 10.1016/j.ophtha.2012.01.052 22521083

