# Supplementary material for: Compliance to contact lens wear and care among Jordanian adults
Source: PLoS One. 2023 Jan 11;18(1):e0280409. doi: 10.1371/journal.pone.0280409 (PMC9833521; doi:10.1371/journal.pone.0280409)
Supplement: S1 Table — (DOCX) [file pone.0280409.s001.docx]

**Compliance to contact lens wear and care among Jordanian adults**

**This survey consists of questions related to contact lens wear and care habits and is designed for completion by adult contact lens wearers in Jordan. The survey should take about 5 minutes to complete. The survey is anonymous and your participation in this study is completely voluntary and you are free to withdraw at any time. Your completion of the survey implies your consent to participate in this research project.**

**If you have questions about this research, please contact:**

**Dr. Yazan Gammoh (**[**y.gammoh@ammanu.edu.jo**](mailto:y.gammoh@ammanu.edu.jo)**; gammohyazan@yahoo.com)**

**Patient number:** __________________________

**Participant demographics:**

Gender: Man /Woman Age: years Smoker Yes/ No

**Residence region:**

North Middle South

**Occupation**:

Professional Management Clerical / Administrative Semi-skilled

Unskilled Retired Student Unemployed

**Educational Level**

None High school Undergraduate Postgraduate

**Refractive Error:**

None Myopia Hyperopia Astigmatism Keratoconus

| **Contact Lens Usage:** | | | | | | | | | | | |
| --- | --- | --- | --- | --- | --- | --- | --- | --- | --- | --- | --- |
| CL type | Soft spherical | | Soft toric | | | | | | Soft cosmetic | Soft multifocal | Rigid Gas permeable |
| CL Power (Spherical Equivalent) | > 5.00 Diopter | | ≤5 Diopter | | | | | | Plano |  | |
| CL Wearing experience (months) |  | | | | | | | | | | |
| Wearing time per day | 1-5 h | | | 6-11 h | | ≥ 12 hours | | | |  | |
| CL wearing modality | Daily disposable | Monthly | | | 3-6 months | | | Yearly | |  | |
| Solution Type/Brand | Multi-purpose | | Hydrogen Peroxide | | | | Saline | | | No solution | |

| **Behaviors** | **Always** | **Frequently** | **Occasionally** | **Rarely** |
| --- | --- | --- | --- | --- |
| Sleeping/napping with CL |  |  |  |  |
| Sharing CL with others |  |  |  |  |
| Swimming/showering with CL |  |  |  |  |
| Topping up solution |  |  |  |  |
| Use of solution after expiry date |  |  |  |  |
| Hands washing before inserting CL |  |  |  |  |
| Rinsing lens with tap water |  |  |  |  |
| Rubbing, rinsing & soaking with prescribed solution |  |  |  |  |
| Cleaning lens case |  |  |  |  |
| Lens case replacement |  |  |  |  |
| Attending after-care visits |  |  |  |  |
